# Regulatory characterisation of the schizophrenia-associated CACNA1C proximal promoter and the potential role for the transcription factor EZH2 in schizophrenia aetiology

**DOI:** 10.1016/j.schres.2018.02.036

**Published:** 2018-09

**Authors:** Kimberley J. Billingsley, Maurizio Manca, Olympia Gianfrancesco, David A. Collier, Helen Sharp, Vivien J. Bubb, John P. Quinn

**Affiliations:** a Department of Molecular and Clinical Pharmacology, Institute of Translational Medicine, University of Liverpool, Liverpool L69 3BX, UK; b Institute of Psychology, Health and Society, University of Liverpool, Liverpool, UK; c Eli Lilly and Company Limited, Windlesham, Surrey, UK

**Keywords:** Genetics, Gene expression, Transcriptomics, Epigenetics, Development

## Abstract

Genomic wide association studies identified the CACNA1C locus as genetically associated with both schizophrenia and bipolar affective disorder. CACNA1C encodes Cav1.2, one of four subunits of L-type voltage gated calcium channels. Variation resides in non-coding regions of CACNA1C which interact with the promoter and are validated expression quantitative trait loci. Using reporter gene constructs we demonstrate the CACNA1C promoter is a major mediator of inducible regulation of CACNA1C activity in the SH-SY5Y neuroblastoma cell line. Exposure of SH-SY5Y cells to lithium and cocaine modulated both the endogenous CACNA1C gene and the promoter in reporter gene constructs. Deletion analysis of the promoter demonstrated the actions of both lithium and cocaine were mediated by the proximal promoter. Initial interrogation of ENCODE ChIP-seq data over the CACNA1C promoter indicated binding of the transcription factor ‘Enhancer of zeste homolog 2’ (EZH2), which was consistent with our data that overexpression of EZH2 repressed CACNA1C promoter reporter gene expression. Array data from the Human Brain Transcriptome demonstrated that EZH2 was highly expressed across the developing brain, but subsequently maintained at low levels after birth and adulthood. RNA-seq data obtained from PD_NGSAtlas, a reference database for epigenomic and transcriptomic data for psychiatric disorders, demonstrated a 3-fold increase in EZH2 expression in the anterior cingulate cortex of individuals with schizophrenia compared to controls. We propose that EZH2 may contribute to schizophrenia risk at two distinct time points either through disruption in development leading to neurodevelopmental changes, or through anomalous reactivation of expression in the adult brain.

## 1 Introduction

It has been proposed that both environmental and genetic factors, termed the gene-environment (G x E) mechanism, contribute to an individual's predisposition to schizophrenia (SCZ) (Ripke et al., 2011). The exact aetiology and genetic mechanism of SCZ is still unclear, however it is likely to involve multiple genes, dysregulation of which may result in altered nervous system responses to environmental challenges (Karlsgodt et al., 2010). Genome-wide association studies (GWAS) offer insight into the genes and mechanisms underpinning SCZ. Our study focuses on the CACNA1C gene which is located on chromosome 12 and codes for the L-type a1c sub-unit of the voltage-dependent calcium channel, Cav1.2. It is the most abundant human neuronal L-type calcium (Ca^2+^) channel and one of the most established and studied SCZ and bipolar affective disorder risk loci (BD) (Obermair et al., 2004). The SCZ and BD associated variation in CACNA1C lies within non-coding regions, which have been reported to be expression quantitative trait loci (eQTLs), thus differential regulation of CACNA1C is presumed to be the mechanism associated with these genetic risks (Eckart et al., 2016). It has been recently shown that these regions work through the CACNA1C promoter; Roussos et al. used chromosome conformation capture (3C-seq) to identify interactions between the most significant CACNA1C SCZ risk SNP (rs1006737) and the CACNA1C promoter in both human post-mortem brain tissue and human induced pluripotent stem cell (hiPSC) derived neurons (Roussos et al., 2014). This promoter-enhancer interaction was further validated using chromosome conformation capture-on-chip (4C-seq) in HEK293 and SK-N-SH cell lines, which determined interaction not only with the strongest SCZ risk SNP (rs1006737) but with the majority of CACNA1C SNPs that are in high linkage disequilibrium (Eckart et al., 2016). This highlighted the importance of the role of the CACNA1C promoter as a key regulatory domain of the gene which could potentially differentially modulate expression in SCZ.

**Supplementary Fig. 1:**
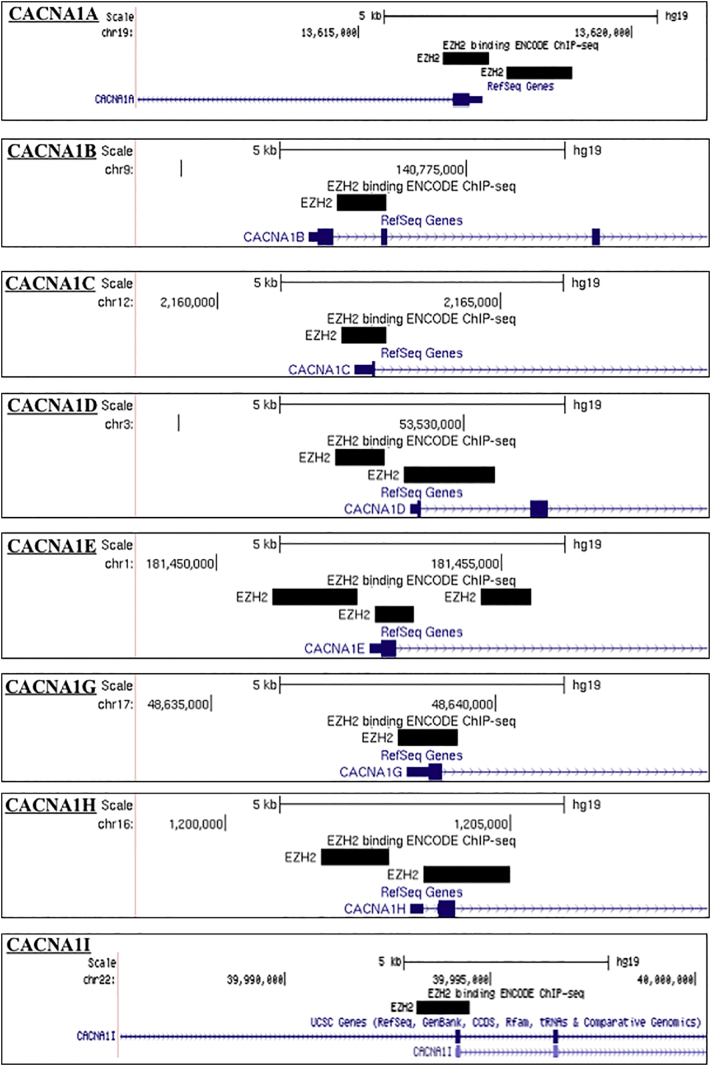

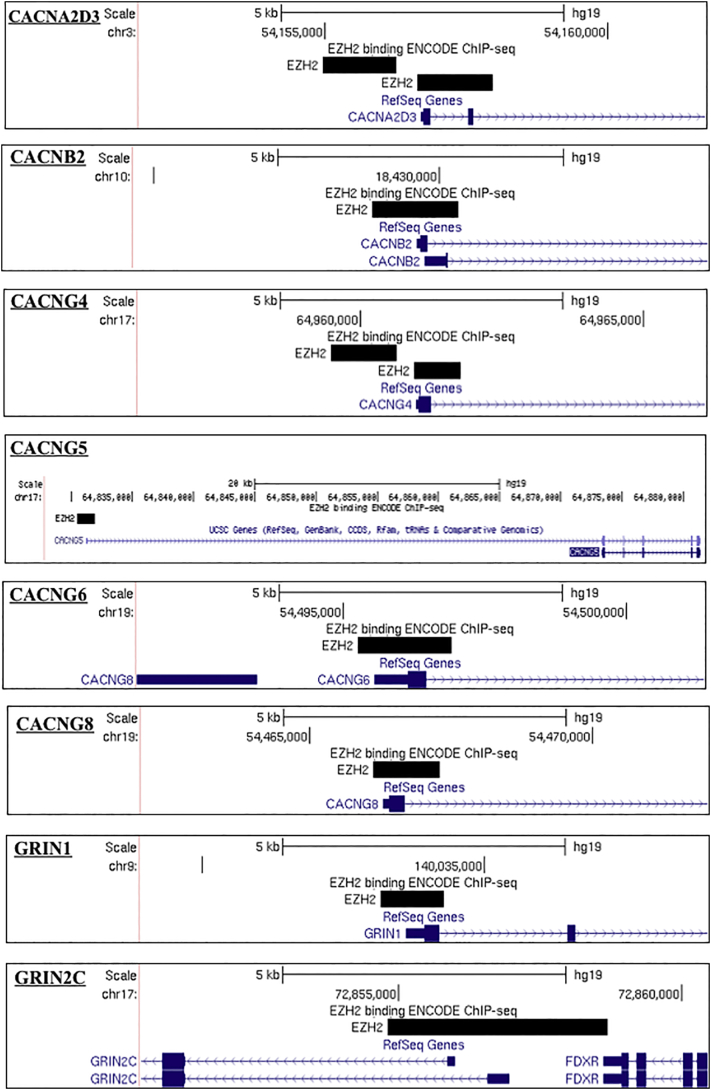

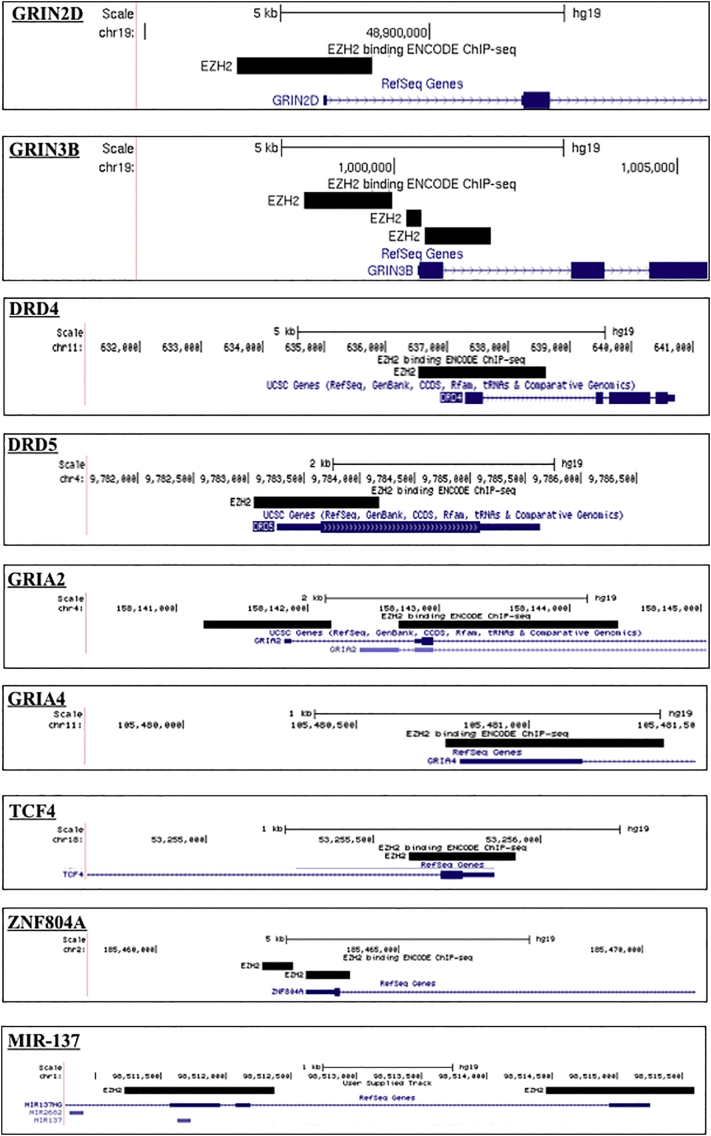
The promoter region of SCZ-associated genes as illustrated in UCSC genome browser (Hg19) highlighting ENCODE transcription factor data (Data version: ENCODE Mar 2012 Freeze) for EZH2 binding sites. Bioinformatic analysis of SCZ-associated genes detected predicted EZH2 promoter binding over major transcriptional starts sites of CACNA1A, CACNA1B, CACNA1C, CACNA1D, CACNA1E, CACNA1G, CACNA1H, CACNA2D3, CACNB2, CACNG4, CACNG6, CACNG8, GRIN1, GRIN2C, GRIN2D, GRIN3B, DRD4, DRD5, GRIA2, GRIA4, TCF4, ZNF804A. As the analysis included all isoforms of the SCZ-associated genes, binding over minor transcriptional start sites was also detected as shown with CACNA1I CACNG5. Thus indicating EZH2 may be regulating specific isoform expression.

The aim of our study was to characterise the promoter region of CACNA1C to further understand this regulatory mechanism. We have previously made use of ENCODE ChIP-seq data to identify and validate regulators of MIR137, a microRNA that has been strongly associated with SCZ specifically (Warburton et al., 2015). Using the same strategy, we identified ‘Enhancer of zeste homolog 2’ (EZH2) as a major regulator of the CACNA1C promoter. EZH2 is a validated target of the SCZ-associated microRNA, MIR137 (Ren et al., 2015; Sun et al., 2015; Szulwach et al., 2010a). Additionaly, interrogation of ENCODE ChIP-seq data demonstrated EZH2 binding at the promoters of MIR137 and multiple genes which are classically associated with SCZ, including glutamate receptors and dopamine receptors (Supplementary Fig. 1). This would suggest two potential mechanisms for the involvement of EZH2 in SCZ, either through direct regulation of expression of calcium channel subunits, glutamate receptors, and dopamine receptors, or indirectly by regulation of a larger subset of SCZ-associated genes through the modulation of MIR137. Bioinformatic analysis of existing datasets also demonstrated high activity of EZH2 during CNS development and perhaps more strikingly in the anterior cingulate cortex of individuals with schizophrenia when compared to controls.

## 2 Methods

### 2.1 Plasmid construction

CACNA1C reporter gene constructs were generated by PCR amplification of the promoter region spanning the transcriptional start site which is denoted by +1; P1 (−149 to +201), P2 (−399 to +201) P3 (−838 to +201). Fragments were amplified from SH-SY5Y DNA using KOD Extreme hot start polymerase (Merck Millipore). The PCR products were subsequently cloned into the pGL3-basic (pGL3b) (Promega) luciferase reporter gene vector using Gibson Isothermal Assembly cloning kit (New England Biolabs). Primer sets and expected product sizes are described in Supplementary Table 1. The constructs were transformed into XL-10 Gold ultracompetent cells (Agilent Technologies). All constructs were confirmed by sequencing (Source Bioscience).

### 2.2 Cell culture

Human neuroblastoma cell line, SH-SY5Y(ATCC CRL-2266), was grown and maintained in a 1:1 mix of Minimal Essential Medium Eagle (Sigma) and Nutrient Mixture F-12 Ham (Sigma), supplemented with 10% foetal bovine serum (ThermoScientific), 100 U/ml penicillin/100 mg/ml streptomycin (Sigma), 1% (v/v) 200 mM l-glutamine (Sigma), and 1% (v/v) 100 mM sodium pyruvate (Sigma). Cells were incubated at 37 °C with 5% CO_2_.

Cells were plated out into 24-well plates for reporter gene transfections or 6-well plates to assay endogenous gene expression. For drug challenge, 10 μM cocaine, 1 mM lithium, or vehicle alone (sterile water) were diluted in appropriate volumes of cell culture media and added to the SH-SY5Y cells for 1 h, removed and fresh media added. For luciferase assays, drug treatments were performed 4-hour post-tranfection.

### 2.3 Luciferase reporter gene assays

SH-SY5Y cells were seeded at approximately 7 × 10^4^ cells per well in 24-well plates. After overnight incubation, cells were 70% confluent and were co-transfected with 1 μg reporter plasmid DNA and 20 ng pMLuc-2 (a thymidine kinase promoter driving renilla luciferase vector used as an internal control; Novagen) using TurboFect Transfection Reagent (ThermoScientific/Fermentas), according to manufacturer's protocol.

Luciferase reporter assays were performed 48-hour post transfection. Luciferase activity of reporter constructs was measured using a Dual Luciferase Reporter Assay System (Promega) using 20 μl lysate from transfected cells according to manufacturer's instructions. Assays were carried out on a Glomax 96-well microplate luminometer (Promega). Measurements were averaged from 4 replicates (3 biological replicates assayed in 4 technical replicates). Statistical analyses were performed using two tailed *t*-tests to calculate significance of the fold change in luciferase expression, compared to the pGL3b backbone vector. To address efficacy of transfection pGL3p and pGL3c luciferase constructs were also included as controls.

### 2.4 RNA extraction and gene expression profiling

Total RNA was extracted from the SH-SY5Y basal and drug challenged cells using Trizol Reagent (Invitrogen), according to manufactures guidelines. RNA concentration was determined using a NanoDrop-8000 spectrometer and 2 μg converted to cDNA using the GoScript RT system. The total reaction volume of 20 μl was diluted 20-fold in nuclease free water and 1 μl of cDNA used per 12 μl PCR reaction using Phusion® High-Fidelity PCR Master Mix (NEB). PCR primers for the CACNA1C transcript targeted exon 1 and 3 of the mRNA sequence: Forward 5′-GAATCAGGTAATCGTCGGCGG-3′ Reverse 5′- GGTTGGAATTGGTGGCGTTGG-3′. PCR primers for the beta-actin (ACTB) transcript targeted exon 2 and 3 of the mRNA sequence:Forward 5′-CACCTTCTACAATGAGCTGCGTGT -3′ Reverse 5′-ATAGCACAGCCTGGATAGCAACGTAC-3′.

### 2.5 EZH2 overexpression assays

For luciferase reporter gene assays, SH-SY5Y cells were co-transfected with a constant amount (0.5 μg) of the CACNA1C promoter construct P1 and increasing amounts of an EZH2 expression plasmid, pCMVHA hEZH2 (range: 0.002 ng–20 ng), a gift from Kristian Helin (Addgene plasmid # 24230). Overall the total amount of DNA (1 μg) was kept constant in the transfection by the addition of the empty pCMVHA backbone. pCMVHA alone was also used as a negative control. Cells were incubated for 48 h before being processed (Bracken et al., 2003).

### 2.6 Bioinformatic analysis

ENCODE ChIP-seq data was accessed through the UCSC Genome Browser (https://genome.ucsc.edu/; genome build GRCh37/hg19). The Human Brain Transcriptome (http://hbatlas.org/) was used to identify levels of EZH2 and CACNA1C expression in multiple regions of the human brain during development and over the lifetime (Kang et al., 2011) (n = 57). The PD_NGSAtlas (available at: http://210.46.85.200/pd_ngsatlas/), a reference database for epigenomic and transcriptomic data for psychiatric disorders, was used to access RNA-seq expression data for EZH2 and CACNA1 genes in the anterior cingulate cortex (BA24) of six individuals with SCZ (as defined by diagnosis based on DSM-IV-TR criteria) and four psychologically healthy controls with no history of axis I conditions (Zhao et al., 2014). All of the subjects were diagnosed by consensus for either SCZ or BD according to DSM-IV-TR criteria and the control samples had no history of an Axis I disorder. The diverse types of clinical characteristics were also collected, including disease status, disease types, age, age of onset, sex and twin status (taken from DOI: https://doi.org/10.1186/s12920-014-0071-z). All patient characteristics that were available from the reference database are detailed in Supplementary Table 2 for the SCZ, BD and control cohorts.

## 3 Results

### 3.1 Luciferase reporter gene expression identifies enhancer and repressor regions within the CACNA1C promoter in the SH-SY5Y neuroblastoma cell line

**Fig. 1:**
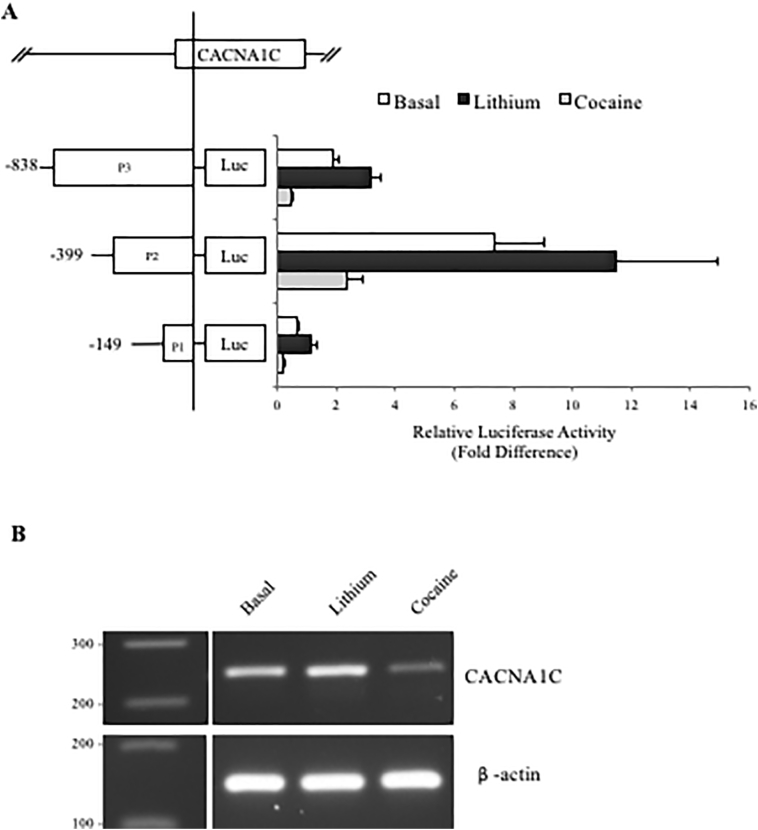
**CACNA1C promoter region constructs in SH-SY5Y cells in a GxE environment contest. A.** Schematic representation of CACNA1C promoter region constructs cloned in the pGL3b (Basic) reporter vector in sense orientation aligned to the CACNA1C gene. Average fold change in luciferase activity supported by the CACNA1C constructs over vector control in SH-SY5Y cells under basal condition and after 1 h treatment with 10 μM cocaine or 1 mM lithium. *N* = *3*. Significant changes in luciferase activity over backbone between experimental conditions are highlighted in table (Supplementary Fig. 2). **CACNA1C promoter region constructs in SH-SY5Y cells in a GxE environment contest. A.** Schematic representation of CACNA1C promoter region constructs cloned in the pGL3b (Basic) reporter vector in sense orientation aligned to the CACNA1C gene. Average fold change in luciferase activity supported by the CACNA1C constructs over vector control in SH-SY5Y cells under basal condition and after 1 h treatment with 10 μM cocaine or 1 mM lithium. *N* = *3*. Significant changes in luciferase activity over backbone between experimental conditions are highlighted in table (Supplementary Fig. 2). **B.** RT-PCR of endogenous CACNA1C mRNA expression in neuroblastoma cell line SH-SY5Y under basal condition and after 1 h treatment with 10 μM cocaine or 1 mM lithium. β-Actin mRNA levels were used for normalisation.

To characterise the promoter of CACNA1C, we generated three reporter gene constructs; P1 (−149 to +201), P2 (−399 to +201) and P3 (−838 to +201) (Supplementary Table 1). The numbering in base pairs was relative to the CACNA1C major transcriptional start site which was denoted as +1. These sequences were based on data from the UCSC genome browser GRCh37/hg19 (Fig. 1A).

**Supplementary Fig. 2:**
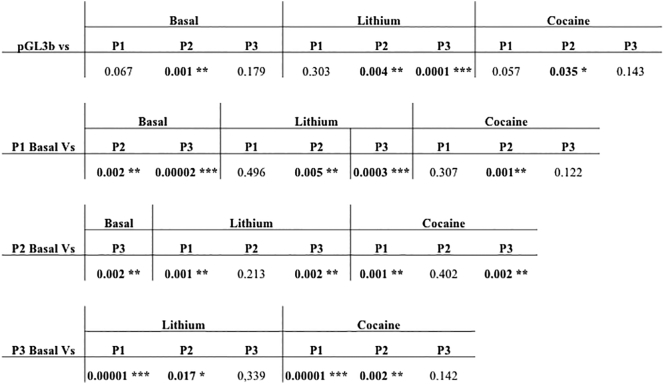
Statistical analysis of the changes in luciferase activity between experimental conditions of the different CACNA1C promoter constructs (*P < 0.05, **P < 0.01, ***P < 0.001).

Reporter gene analysis in the SH-SY5Y neuroblastoma cell line demonstrated that the CACNA1C promoter contained both positive and negative regulatory domains within the sequences examined. Both P2 (##P < 0.01) and P3 (###P < 0.001) supported significant increases in reporter gene expression over the shortest promoter fragment tested, P1 (minimal promoter fragment used, hereafter referred to as minimum promoter). However, P2 supported a greater 3.5-fold increase in reporter activity compared to P3 (Fig. 1A and Supplementary Fig. 2; ##P < 0.01) indicating that a repressor domain was present between bases −839 to −399. Similarly, the difference in luciferase expression between P2 and P1 indicated a strong positive regulator between −399 and −149.

### 3.2 Gene expression is directed through the minimal promoter and is modulated in a stimulus-inducible manner

The SH-SY5Y cells were treated with a stimulant (cocaine) or a mood stabiliser (lithium) to determine if transcriptional activity at the CACNA1C locus was regulated in a stimulus-inducible manner. We and others have previously used these drugs, at the concentrations specified in the current study, to modulate signaling pathways in SH-SY5Y cells, including the MIR137 gene network (Warburton et al., 2015). Exposure to 1 mM lithium resulted in increased expression from all three CACNA1C promoter constructs. This was in contrast to exposure to 10 μM cocaine which resulted in repression of all reporters. However, the drug challenge data indicated that P1, the minimal promoter region, directed similar fold changes in modulation of reporter gene expression to both P2 and P3 (Fig. 1A). This would be consistent with a major modulator of response to lithium and cocaine being located within (−149 to +201), which spans the transcriptional start site.

Expression of the endogenous CACNA1C gene in SH-SY5Y cells was analysed by RT-PCR and was found to be modulated by both drugs similar to that of the reporter constructs (Fig. 1B); lithium increased, whilst cocaine decreased expression (expression normalised to the housekeeping gene β-actin).

### 3.3 The transcription factor “Enhancer of zeste homolog 2” represses transcription from the CACNA1C minimal promoter

**Fig. 2:**
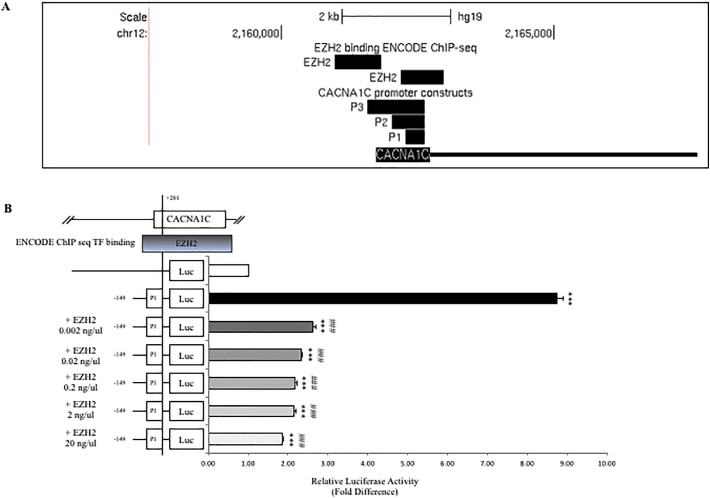
**The effect of EZH2 overexpression on the CACNA1C minimal promoter region in SH-SY5Y cells. A.** CACNA1C promoter region as illustrated in UCSC genome browser (GRCh37/hg19) highlighting EZH2 binding sites predicted by ENCODE ChIP-seq data (Data version: ENCODE Mar 2012 Freeze). **B**. Schematic representation of CACNA1C minimal promoter region construct (P1) cloned in the pGL3b (Basic) reporter vector in sense orientation. On the left side are shown the different concentrations of EZH2 plasmid transfected, on the right is shown the average fold change in luciferase activity supported by the CACNA1C construct when co-transfected with the EZH2 plasmid. *N* = *3*. *Significant changes in luciferase activity over backbone control. #Significant changes in luciferase activity between experimental conditions (***/### P < 0.001).

**Supplementary Fig. 3:**
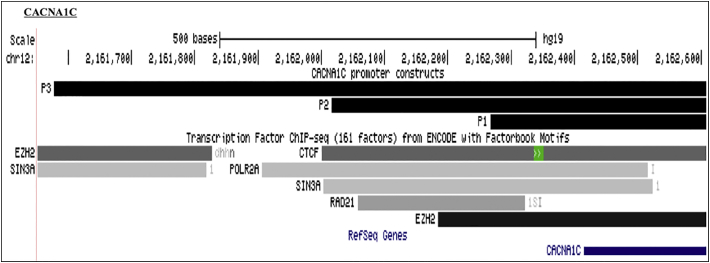
The promoter region of CACNA1C illustrated in UCSC genome browser (Hg19) highlighting ENCODE transcription factor data (Data version: ENCODE Mar 2012 Freeze) for EZH2, POLR2A SIN3A, CTCF and RAD21 binding sites over the major transcriptional start site.

ENCODE ChIP-seq data identified the transcriptional repressor EZH2, a validated target for the SCZ-associated microRNA, MIR137 (Szulwach et al., 2010a), over the CACNA1C promoter (Fig. 2A). There were several predicted binding sites for EZH2 in this locus, two of which fell in the regions investigated by our reporter gene studies. ENCODE data (Supplementary Fig. 3) also identified promoter binding sites for RNA polymerase II, SIN3A, CTCF, and RAD21. We analysed the effect of overexpression of EZH2 on the minimum promoter reporter gene construct in SH-SY5Y cells, which resulted in repression of reporter gene activity at all concentrations tested (Fig. 2B; ***/### P < 0.001). This was consistent with the ENCODE data predicting that EZH2 would bind to the region encompassed by the P1 construct (−149 to +201).

Further examination of ENCODE ChIP-seq data demonstrated EZH2 binding at the MIR137 promoters, as well as at the promoters of 14 of the 26 genes encoding calcium voltage-gated channel subunits (54%), and multiple genes from the glutamate and dopamine receptor subfamilies. Further, we identified EZH2 binding at the promoters of SCZ GWAS genes, TCF4 and ZNF804A (Supplementary Fig. 1). As EZH2 was identified at the promoters of multiple genes and gene families which are known to be strongly associated with SCZ, this would suggest that modulation of EZH2 could contribute to SCZ risk either through direct regulation at the promoters of SCZ-associated genes, and/or through altered regulation of a wider SCZ-associated network by modulating MIR137 expression.

### 3.4 A role for EZH2-mediated calcium channel regulation in brain development and psychiatric conditions

**Fig. 3:**
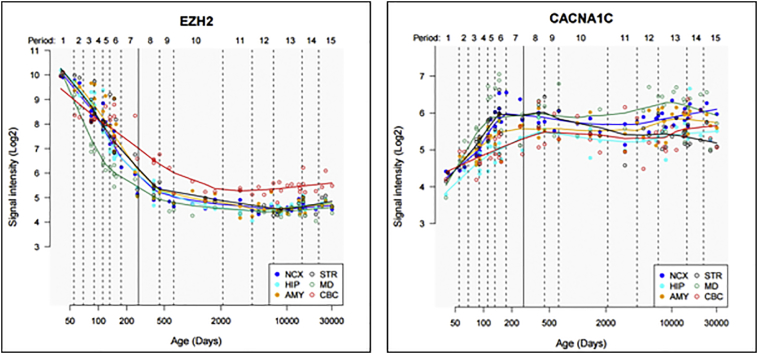
Expression profile of EZH2 and CACNA1C in the developing and adult human brain. Gene expression data from the Human Brain Transcriptome, encompassing timepoints from embryonic development through to late adulthood, shows an inverse correlation between EZH2 and CACNA1C expression, particularly during development. EZH2 expression is at its highest 4–8 weeks post-conception (Period1) when embryonic stages of brain development occur, during the same period CACNA1C expression is at its lowest, with a Log2 signal intensity of 10 and 4, respectively. As EZH2 expression decreases globally across the brain during development, CACNA1C expression increases, with expression of both genes plateauing around the time of birth (solid line). This would suggest a role for EZH2 in the regulation of CACNA1C across the developing human brain. This dataset combined information on gene expression from 16 brain regions, comprised of 11 regions of the neocortex (NCX), and one region from the hippocampus (HIP), amygdala (AMY), striatum (STR), mediodorsal nucleus of the thalamus (MD), and the cerebellar cortex (CBC) for all n = 57. Periods 1–15 are based on age of the samples and detailed in Kang et al. (http://www.nature.com/nature/journal/v478/n7370/abs/nature10523.html), with Periods 1–7 representing embryonic and foetal stages, and stages 8–15 referring to birth to 82 years of age. A solid vertical line indicating birth separates these two main phases. In support of this, gene expression data from the BrainCloud (http://www.libd.org/braincloud) shows the same inverse correlation of expression for both genes during brain development in human dorsolateral prefrontal cortex postmortem tissue (data not shown).

**Fig. 4:**
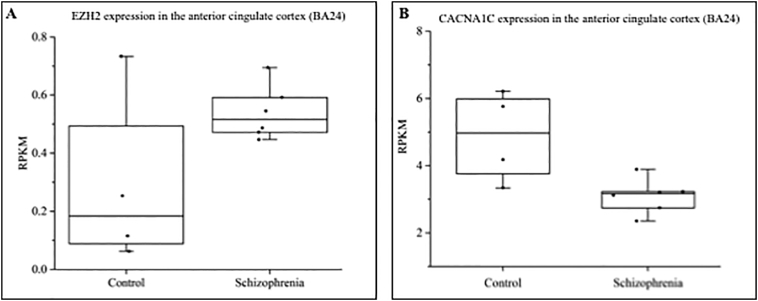
Expression of EZH2 and the CACNA1 gene family in the anterior cingulate cortex (BA24) of individuals with a diagnosis of schizophrenia versus controls. (A) Publicly available RNA-seq data from the PDNGS_Atlas showed a median 3-fold increase in EZH2 expression in the anterior cingulate cortex of six individuals with schizophrenia when compared to four control individuals (RPKM = Reads per kilobase of transcript per million mapped reads). EZH2 expression was seen to be typically maintained at low levels in the adult brain, with a median expression of 0.18 RPKM in controls. However, in the brains of individuals with schizophrenia in this data set, median EZH2 expression was increased almost 3-fold to 0.52 RPKM. (B) In the same samples a corresponding 1.6-fold decrease of CACNA1C was found in individuals with schizophrenia compared to controls.

Our data indicated a role for EZH2 in the regulation of CACNA1C and other calcium channel subunits, which could be central to molecular mechanisms involved in SCZ given the GWAS association of CACNA1C, and the observation that EZH2 is a validated target and predicted regulator of the SCZ-associated microRNA, MIR137 (Ren et al., 2015; Sun et al., 2015; Szulwach et al., 2010a) (Supplementary Fig. 1). To gain insight into the key time points at which this pathway might be active, we characterised the changing expression profiles of EZH2 and CACNA1C in the developing and adult brain using the Human Brain Transcriptome resource (http://hbatlas.org/) (Fig. 3). No data for MIR137 was available in this data set. We identified that the highest level of expression of EZH2 in the brain was at 4 to 8 weeks post-conception (Period 1). Conversely, at this time point we saw that CACNA1C expression was at its lowest level compared to the remainder of the lifetime. EZH2 expression decreased globally across the brain during foetal development, but plateaued shortly after birth (solid line) at 6 to 12 months of age (corresponding to Period 9; Fig. 4). EZH2 expression was thereafter maintained at low levels across the brain throughout the remainder of the lifetime. As EZH2 decreased throughout the course of foetal development, CACNA1C expression increased in a linear fashion until shortly before birth, when expression plateaued in most brain areas at around the late mid-foetal stage (19 to 24 weeks post-conception). Thus during the prenatal period of embryonic and foetal development, an inverse correlation between EZH2 and CACNA1C expression was apparent. This would be consistent with the role of EZH2 in regulating the expression of developmental genes in the early brain (Hirabayashi et al., 2009; Qi et al., 2013; Ronan et al., 2013; Sher et al., 2012; Zhao et al., 2015). We therefore postulate that differential or aberrant modulation of EZH2 expression during foetal brain development may contribute to the neurodevelopmental aspects of SCZ risk.

**Supplementary Fig. 4:**
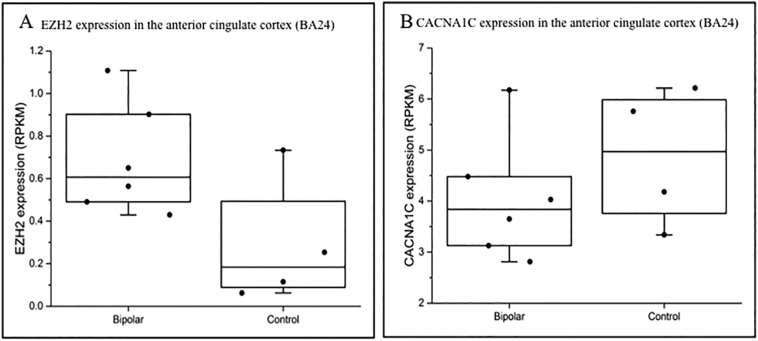
Expression of EZH2 and CACNA1C in the anterior cingulate cortex (BA24) of individuals with a diagnosis of Bipolar Disorder versus controls. (A) Publicly available RNA-seq data from the PDNGS_Atlas showed a median 3.3-fold increase in EZH2 expression in the anterior cingulate cortex of six individuals with Bipolar Disorder when compared to four control individuals (RPKM = Reads per kilobase of transcript per million mapped reads). EZH2 expression was seen to be typically maintained at low levels in the adult brain, with a median expression of 0.2 RPKM in controls. However, in the brains of individuals with Bipolar Disorder in this data set, median EZH2 expression was increased almost 3-fold to 0.6 RPKM. (B) In the same samples a corresponding 0.7-fold decrease of CACNA1C was found in individuals with Bipolar Disorder compared to controls.

After birth, EZH2 expression is maintained at low levels throughout the brain across the rest of the lifetime (Fig. 3). Therefore, a second mechanism by which EZH2 may contribute to SCZ risk is through anomalous reactivation in the adult brain. To test this hypothesis, we utilised RNA-seq expression data from the PD_NGSAtlas which hosts publically available epigenomic and transcriptomic data from control and SCZ brain samples. We compared RNA-seq data from the anterior cingulate cortex and Brodmann area 24 (BA24) of six individuals with a diagnosis of SCZ and four controls. We saw a median 3-fold increase in EZH2 expression in SCZ versus control samples (Fig. 4A) which correlated with a 1.6-fold downregulation of CACNA1C in individuals with SCZ compared to controls (Fig. 4B). Noting that variation in CACNA1C can also confer a risk for BD, we conducted a similar analysis with the PD_NGSAtlas data available for the BD cohort. Equally this showed that in the anterior cingulate again there was an inverse relationship between EZH2 and CACNA1C (Supplementary Fig. 4). Unfortunately, there was no data available for EZH2 expression in the hippocampus for either cohorts.

## 4 Discussion

CACNA1C has been strongly implicated in the aetiology of SCZ and BD, not only due to the genome-wide significant SCZ-associated SNPs in the introns of the gene (Hamshere et al., 2013; Psychiatric, 2011; Schizophrenia Working Group of the Psychiatric Genomics, 2014), but also in SCZ because it is a known target of MIR137 (Collins et al., 2014; Szulwach et al., 2010b). Differential CACNA1C expression has been associated with SCZ risk (Bigos et al., 2010). We hypothesised that the promoter region of the gene would play an important role, as one of the CACNA1C enhancer regions containing a SCZ associated risk loci (SNP) has been shown to interact with the CACNA1C promoter (Eckart et al., 2016; Roussos et al., 2014). Using CACNA1C promoter deletion constructs to analyse the modulation of a reporter gene in SH-SY5Y neuroblastoma cells, we identified a positive regulator within the region of −399 to −149 at the promoter, and a repressor in the region of −838 to −399 (Fig. 1A). All three of the reporter constructs utilised in this study responded to both cocaine and lithium, which mirrored changes in endogenous CACNA1C expression (positively in response to lithium and negatively in response to cocaine). The responses of all three of our promoter constructs were equivalently modified by these drugs indicating that the promoter region (−149 to +201) common to all 3 constructs was mostly likely mediating this response to the challenge (Fig. 1). Bioinformatic analysis using ENCODE ChIP-seq data was used to address potential transcription factor binding over the CACNA1C transcriptional start site and this demonstrated binding of the transcription factor EZH2 at this location (Fig. 2A). Like CACNA1C itself, EZH2 is a validated target of MIR137. EZH2 over expression significantly repressed reporter gene expression from the CACNA1C minimal promoter P1 construct (−149 to +201) in SH-SY5Y cells (Fig. 2B), thus confirming EZH2 as a regulator of this CACNA1C promoter region.

The action of EZH2 upon transcription of CACNA1C in vivo was addressed using freely available expression data sets for both human development and adult CNS. Analysis of data from the Human Brain Transcriptome demonstrated that EZH2 was predominantly active in the brain during development (Fig. 3), with the highest level of expression seen in embryonic stages. Globally, an inverse correlation between EZH2 and CACNA1C expression across the developing brain was found, thus we postulate that EZH2 may be one of the regulators of the expression of CACNA1C during the developmental process. This could contribute to the neurodevelopmental risk factors for SCZ (Brown, 2012; Estes and McAllister, 2016; Kim et al., 2015; Scheinost et al., 2017), and is consistent with EZH2 acting as an important modulator of gene expression in the developing brain. For example, mouse models demonstrated the role for EZH2-mediated regulation of Reelin-controlled migration of neurons during the development of the cerebral cortex, with knockdown of EZH2 resulting in impaired migration (Zhao et al., 2015). Similarly, Pereira et al. demonstrated that EZH2 was critical in controlling the rate of development in cortical progenitor cells, with changes in EZH2 expression altering the balance between cell self-renewal and differentiation, resulting in altered timing of cortical development (Pereira et al., 2010). In the adult, we identified a 3-fold increase in EZH2 expression in the anterior cingulate cortex of individuals with a diagnosis of SCZ when compared to controls (Fig. 4A), which correlated with downregulated expression of CACNA1C in the brains of the same individuals with SCZ (Fig. 4B). We also observed a similar inverse correlation of EZH2 and CACNA1C in BD. In addition to CACNA1C, modulation of EZH2 in SCZ is likely to cause dysregulation of a larger set of SCZ-associated targets including SCZ GWAS genes, MIR137, TCF4, and ZNF804A, as well as multiple members of the calcium channel, glutamate receptor, and dopamine receptor gene families (Supplementary Fig. 1).

**Fig. 5:**
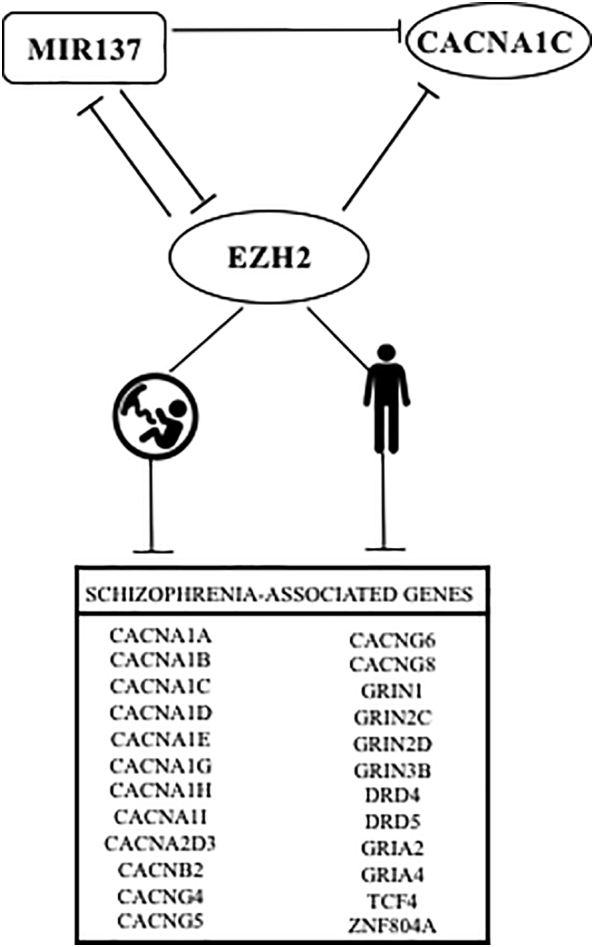
Proposed mechanism for MIR137-EZH2 mediated regulation of a larger schizophrenia gene network. CACNA1C and EZH2 are both validated targets of the schizophrenia-associated microRNA, MIR137. We have validated EZH2 as a modulator of expression from the CACNA1C promoter, and demonstrated EZH2 binding at the promoter of many schizophrenia-associated genes, including MIR137, through ENCODE ChIP-seq. This would suggest a regulatory loop between EZH2 and MIR137, as well as identifying EZH2-mediated regulation of a distinct schizophrenia-associated gene set. We therefore propose two distinct mechanisms through which EZH2 may regulate schizophrenia-associated genes, either through directly binding to the promoters of its schizophrenia-associated target genes, or through the regulation of MIR137, thereby resulting in indirect regulation of the MIR137 target gene set. We observed high expression of EZH2 across the foetal brain which was then maintained at low levels after birth. However, as our model would suggest, we found increased EZH2 expression in the anterior cingulate cortex of individuals with a diagnosis of schizophrenia.

Based upon transcriptome data we propose a role for EZH2 in the aetiology of SCZ at two separate timepoints; firstly in regulating a downstream SCZ-associated gene set during brain development, and/or secondly in modulating levels of SCZ-associated targets in the adult brain. Modulation of EZH2 expression at either timepoint, or indeed both, may result in dysregulation of SCZ-associated targets, altering CNS development or causing dysregulation of genes in the adult brain. Further, as EZH2 is a validated target of MIR137, EZH2 modulation may result in the dysregulation of SCZ-associated genes; either through direct modulation via binding at the promoter of SCZ-associated genes (Supplementary Fig. 1), or through regulating the expression of MIR137 affecting the SCZ-associated MIR137 target genes. We note here that MIR137 has not been associated with BD and therefore we may be beginning to see differences in related pathways overlapping SCZ and BD. The latter allows for a model in which MIR137 and EZH2 levels are potentially in a homeostasis loop modulating the expression of a larger network of genes regulating Ca^2+^ influx and glutamatergic signaling in the regulation of axonal growth and guidance (Rosenberg and Spitzer, 2011) specific for SCZ (Fig. 5).

We have not addressed directly the role of the CACNA1C schizophrenia risk SNP (rs1006737) to modulate the promoter nor its interaction with EZH2 in this communication. However the role of EZH2 on the promoter suggests there might be convergence of function and that we have highlighted. This SNP has been strongly associated with both SCZ and BD, however it is well documented that there is a pleiotropic effect of this polymorphism, again supporting the idea that the functional importance of the polymorphism is disease-specific and therefore implies a disease specific regulatory pathway might be involved. Our demonstration of a potential pathway in Fig. 5 involving CACNA1C, EZH2 and MIR137 may be one pathway to explore the different action of this SNP in distinct brain regions and conditions. This transcriptional model will be modified by tissue-specific and environmental parameters to which the cell is exposed, in addition to an individual's genetic variation, which is likely to influence susceptibility to SCZ.

The following are the supplementary data related to this article.

**Supplementary Table 1:**
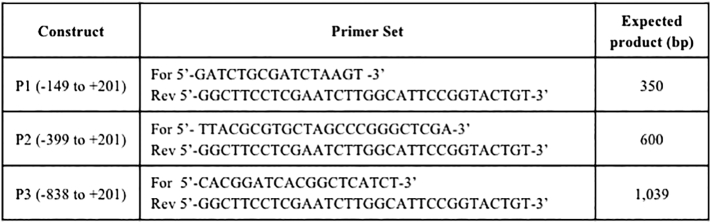
Primer sets and expected product size (bp) for each of the three CACNA1C promoter fragments.

**Supplementary Table 2:**
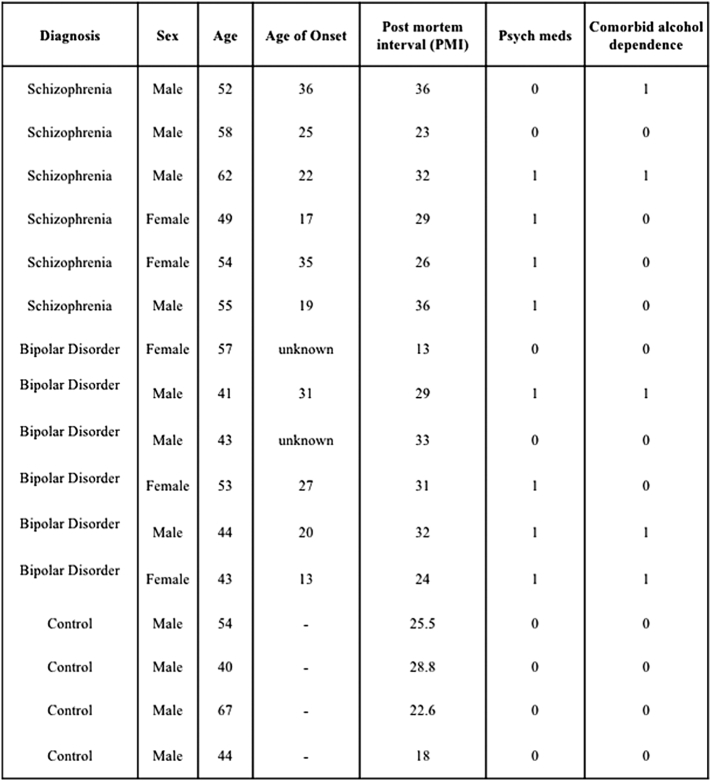
Characteristics of the SCZ, BD and Control cohorts.

## Funding

Olympia Gianfrancesco was the recipient of BBSRC Case Studentship ID 119131.

## Conflict of interest

David A Collier is a full-time employee and stockholder of Eli Lilly and Company.
